# Hidradenoma papilliferum of the caruncle: a case report

**DOI:** 10.1093/jscr/rjag789

**Published:** 2026-09-03

**Authors:** Amer Alemam, Husam Khaleel, Ghadeer Almuhaisen, Ayman Musleh, Hashem Abu Serhan

**Affiliations:** Department of Ophthalmology, Eye Specialty Hospital, Al-Madina Al-Monawara St., Tla Al-Ali, PO Box 1494, Amman 11821, Jordan; Department of Ophthalmology, Eye Specialty Hospital, Al-Madina Al-Monawara St., Tla Al-Ali, PO Box 1494, Amman 11821, Jordan; Department of Pathology, School of Medicine, Mutah University Mutah, Mutah Street, PO Box 7, Al-Karak 61710, Jordan; Department of Ophthalmology, Eye Specialty Hospital, Al-Madina Al-Monawara St., Tla Al-Ali, PO Box 1494, Amman 11821, Jordan; Department of Ophthalmology, Hamad Medical Corporation, Al Rayyan, Zone 37, Street 15, PO Box 3050, Doha, Qatar

**Keywords:** hidradenoma papilliferum, caruncle, benign adnexal tumor, ocular surface lesion

## Abstract

Hidradenoma papilliferum (HAP) is a rare benign adnexal tumor of apocrine differentiation, typically occurring in the vulvar or perianal region of women. We report an exceptionally rare case of HAP arising from the lacrimal caruncle, with only one similar case previously documented in the ophthalmic literature. A 61-year-old woman presented with a 3-month history of a painless, vascularized reddish mass over the right caruncle. Complete surgical excision was performed without complications. Histopathological examination revealed a well-circumscribed lesion with papillary and glandular architecture, fibrovascular cores, and a characteristic bilayered epithelium with apocrine features, consistent with HAP and without evidence of malignancy. This case highlights the importance of considering HAP in the differential diagnosis of caruncular tumors and confirms surgical excision as curative.

## Introduction

The skin is the largest and most functionally diverse organ of the human body, containing numerous sweat glands distributed across most of the body’s surface. These sweat glands function through either apocrine secretion, where portions of the outer cell membrane pinch off, or eccrine secretion, which releases liquid without cellular disintegration [1]. The caruncle is a small, fleshy, ovoid structure of modified skin found attached to the inferomedial side of the plica semilunaris [2]. It contains sebaceous glands and fine, colorless hairs [2].

Hidradenoma papilliferum (HAP), also known as papillary hidradenoma, is a rare but benign dermal papulonodular tumor of apocrine differentiation. It is predominantly found in the vulvar/perianal region of females [3]. We report an exceptionally rare case of HAP occurring at the caruncle; a highly unusual location for this lesion, with only one prior case documented in ophthalmic literature with similar location.

## Case report

**Figure 1:**
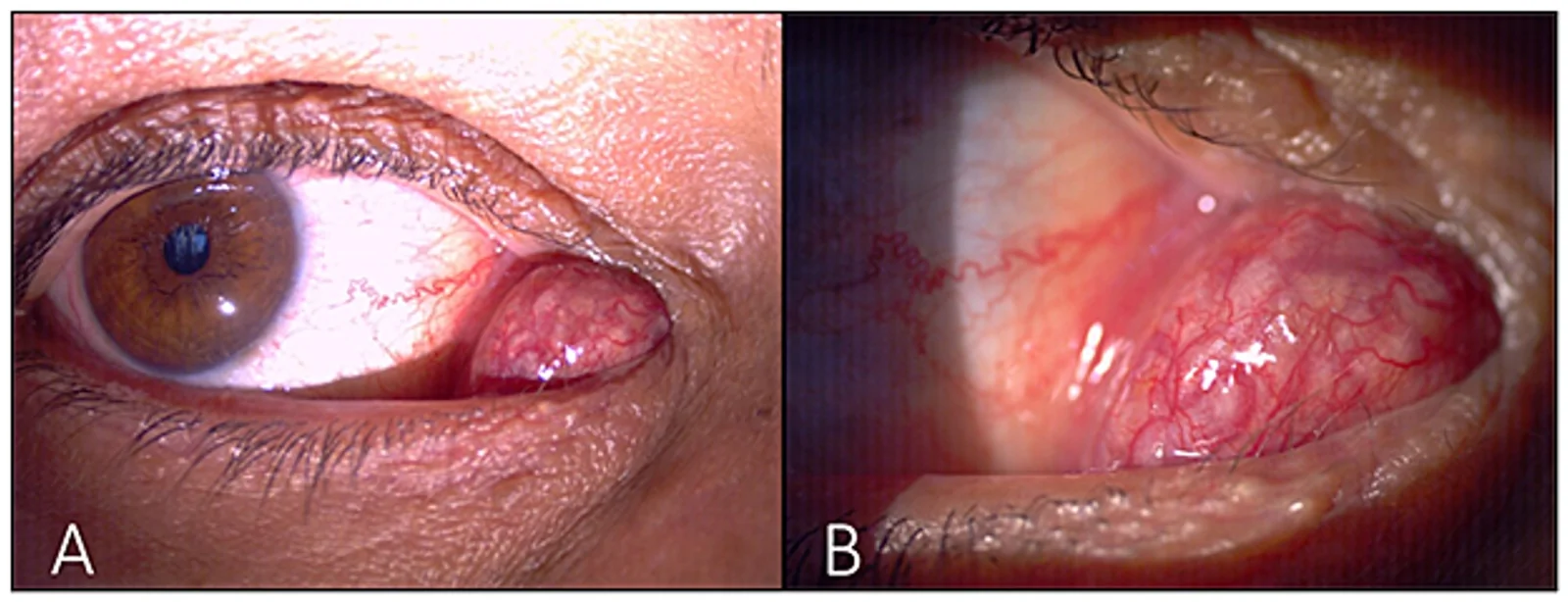
(A) The clinical appearance of the right eye caruncular mass, (B) a magnified image of the mass.

A 61-year-old female patient presented to the general ophthalmology clinic complaining of uncomfortable right nasal swelling. The patient is known to have well-controlled diabetes mellitus type 2. She denied any trauma and said that she first noticed the swelling around 3 months prior to presentation. On slit-lamp examination, a nonmobile, firm, heavily vascularized, reddish mass, measuring 10.0 × 15.0 mm, was found covering the caruncle of the right eye (Fig. 1). The remaining evaluation was unremarkable. An oculoplastic surgeon was consulted, and a complete excision of the mass was performed under local anesthesia using no-touch surgical technique without complications. The mass was sent for histopathological examination.

**Figure 2:**
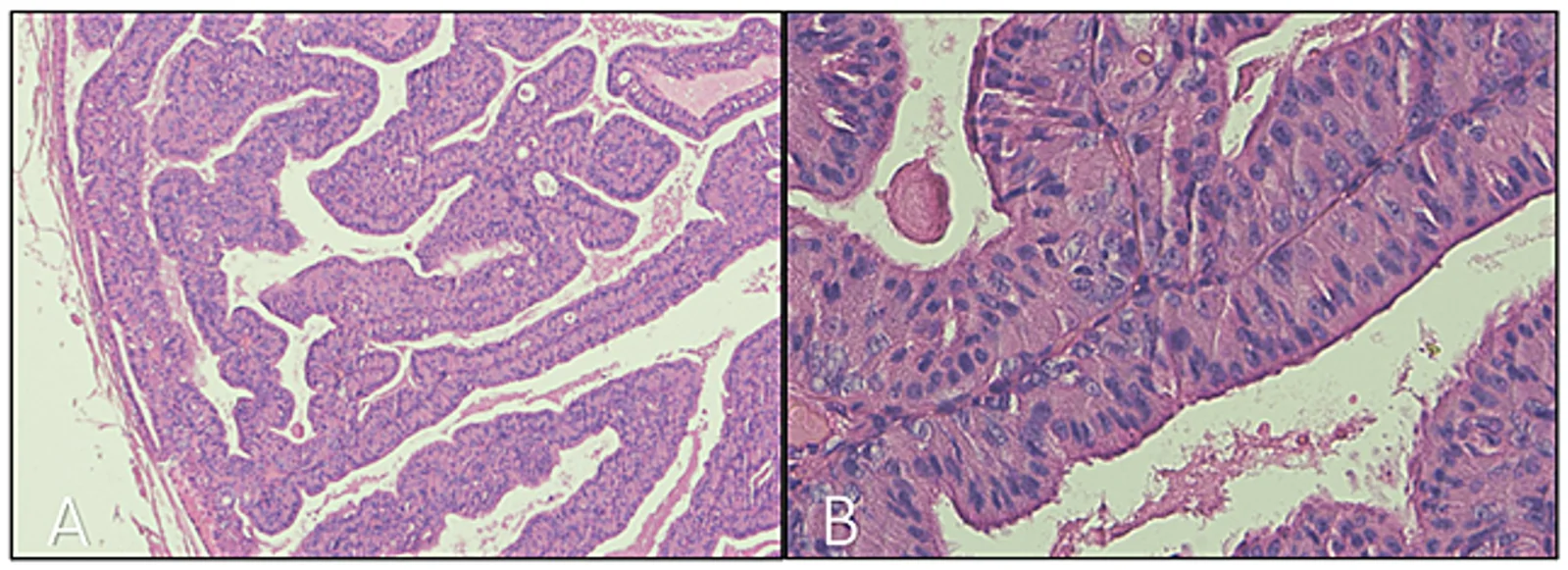
Microscopic examination of the tumor. (A) A well-circumscribed proliferation of arborizing papillary structures (H&E 40×). (B) Higher-magnification picture highlights the bilayered epithelium of those papillae; luminal columnar cells and underlying myoepithelial layer (H&E, 400×).

The histopathological images exhibit characteristic features of HAP. The lesion is well-circumscribed and shows a papillary and glandular architecture, with multiple complex papillary fronds and tubulo-papillary structures extending into cystic lumina. The stroma contains fibrovascular cores, supporting the epithelial proliferation. The epithelial component is bilayered, consisting of a luminal layer composed of tall columnar cells with eosinophilic cytoplasm and round to oval nuclei, and a basal layer containing small cuboidal to myoepithelial cells with darker, basophilic nuclei. The nuclear morphology is bland, with minimal pleomorphism and low mitotic activity, confirming its benign nature. Areas of eosinophilic secretory material in luminal spaces further confirm apocrine differentiation. No significant inflammatory infiltrate or desmoplasia is observed (Fig. 2).

## Discussion

First described by Werth in 1878, HAP is a benign tumor of apocrine differentiation that affects primarily the anogenital region of middle-aged females [3]. However, these tumors can occasionally be found in different locations across the human body and are thereby labeled ectopic. Ectopic HAP tumors are typically found in older patients, usually one to two decades older than the average age range of those with anogenital HAP [4]. Unlike anogenital HAP, nearly half of the reported cases of ectopic HAP occur in men [4]. Ectopic HAP most commonly occurs in the head and neck region, accounting for 60% of cases [4, 5]. Although it is well established that the eyelid skin and caruncle contain apocrine sweat glands [2], reports of ectopic HAP in ophthalmic literature remain exceedingly rare. Only a few documented cases have involved the eyelids [6–8], eyebrow [9, 10], and even a single instance of orbital involvement [11]. To the best of our knowledge, its presentation as a caruncular lesion has been reported only once [12].

HAP is a benign tumor with no risk of recurrence if completely excised. Clinically, it appears as a discreet, solitary nodule of variable size. On histopathological evaluation, the tumor typically exhibits a partially cystic structure with both papillary and glandular components, as observed in our case. The presence of a myoepithelial cell layer and secretory granules serves as a key distinguishing factor between apocrine and eccrine lesions [7]. While the risk of malignant transformation remains low, wide local excision is the preferred treatment.

In conclusion, HAP of the lacrimal caruncle represents an exceptionally rare entity that may clinically mimic more common caruncular lesions. Awareness of this diagnosis is important for ophthalmologists and pathologists when evaluating vascularized adnexal masses in this location. Histopathological examination remains essential for definitive diagnosis, and complete surgical excision is both diagnostic and curative. Reporting such rare presentations contributes to the existing literature and helps broaden the differential diagnosis of caruncular tumors.
